# Interaction of ascaridole, carvacrol, and caryophyllene oxide from essential oil of Chenopodium ambrosioides L. with mitochondria in *Leishmania* and other eukaryotes

**DOI:** 10.1002/ptr.6097

**Published:** 2018-04-19

**Authors:** Lianet Monzote, Gerald Geroldinger, Matthias Tonner, Ramón Scull, Sritama De Sarkar, Sophie Bergmann, Markus Bacher, Katrin Staniek, Mitali Chatterjee, Thomas Rosenau, Lars Gille

**Affiliations:** ^1^ Institute of Pharmacology and Toxicology, Department of Biomedical Sciences University of Veterinary Medicine Vienna Vienna Austria; ^2^ Department of Parasitology Institute of Tropical Medicine “Pedro Kourí” Havana Cuba; ^3^ Department of Chemistry, Institute of Pharmacy and Food Havana University Havana Cuba; ^4^ Department of Pharmacology Institute of Postgraduate Medical Education & Research Kolkata India; ^5^ Department of Chemistry, Division of Chemistry of Renewables University of Natural Resources and Life Sciences Tulln Austria

**Keywords:** ascaridole, carvacrol, caryophyllene oxide, Chenopodium ambrosioides L, Leishmania, mitochondria

## Abstract

The antileishmanial activity of the essential oil (EO) from Chenopodium ambrosioides L. has been demonstrated in vitro and in animal models, attributed to the major components of the EO. This study focused on the effects of the three major EO compounds carvacrol, caryophyllene oxide (Caryo), and the antileishmanial endoperoxide ascaridole (Asc) on mitochondrial functions in *Leishmania tarentolae* promastigotes (LtP). EO and Caryo were able to partially inhibit the leishmanial electron transport chain, whereas other components failed to demonstrate a direct immediate effect. Caryo demonstrated inhibition of complex III activity in LtP and in isolated complex III from other species. The formation of superoxide radicals was studied in *Leishmania* by electron spin resonance spectroscopy in the presence of iron chelators wherein selected compounds failed to trigger a significant immediate additional superoxide production in LtP. However, upon prolonged incubation of *Leishmania* with Asc and especially in the absence of iron chelators (allowing the activation of Asc), an increased superoxide radical production and significant impairment of mitochondrial coupling in *Leishmania* was observed. Prolonged incubation with all EO components resulted in thiol depletion. Taken together, the major components of EO mediate their leishmanicidal activity via different mitochondrial targets and time profiles. Further studies are required to elucidate possible synergistic effects of carvacrol and Asc and the influence of minor compounds.

**Abbreviations**
AA: antimycin A
Asc: ascaridole
BH‐*bc*_1_: complex *bc*_1_ from submitochondrial particles of bovine heart
BH‐SMP: submitochondrial particles from bovine heart
BSA: bovine serum albumin
Car: carvacrol
Caryo: caryophyllene oxide
CCCP: carbonyl cyanide‐*m*‐chlorophenylhydrazone
CMFDA: 5‐chloromethylfluorescein diacetate
CMH: 1‐hydroxy‐3‐methoxycarbonyl‐2,2,5,5‐tetramethylpyrrolidine‐HCl
CM^●^: radical of CMH
cyt *c*^3+^: cytochrome *c* ^3+^
DCPIP: 2,6‐dichlorophenol–indophenol
DFO: desferrioxamine mesylate
DMSO: dimethyl sulfoxide
DTPA: diethylenetriaminepentaacetic acid
DTT: dithiothreitol
dUQ: decylubiquinone
dUQH_2_: decylubiquinol
EDTA: ethylenediamine tetraacetic acid
EO: essential oil from *Chenopodium ambrosioides* L.
ESR: electron spin resonance
ETC: electron transport chain
IC_50_: median inhibitory concentration
LaP: *Leishmania amazonensis* promastigotes
LtP: *Leishmania tarentolae* promastigotes
LtP‐Mit: mitochondrial fraction from *Leishmania tarentolae* promastigotes
NADH: reduced nicotinamide–adenine dinucleotide
NMR: nuclear magnetic resonance
Oligo: oligomycin
PBS: phosphate‐buffered saline
RCR: respiratory control ratio
ScY: *Saccharomyces cerevisiae* yeast
ScY‐*bc*_1_: complex *bc*_1_ from submitochondrial particles of *Saccharomyces cerevisiae*
ScY‐SMP: submitochondrial particles from *Saccharomyces cerevisiae*
SMP: submitochondrial particles
Tris: tris(hydroxymethyl)aminomethane
YEM: yeast extract medium

## 1 INTRODUCTION

Chenopodium ambrosioides L. is an aromatic herb native to Central and South America. It has been distributed throughout the tropical parts of the world and is considered as invasive (Duke, Bogenshutz, du‐Cellier, & Duke, 2002; Trivellato‐Grassi et al., 2013). The plant is annual or perennial; it can grow up to 1 m in height, and one of its main characteristics is the strong aromatic odor. It has been employed by empirical herbalists and healers, particularly against parasites (Franca, Lago, & Marsden, 1996; Quinlan, Quinlan, & Nolan, 2002). Some significant biological properties have been demonstrated, including antitumor (Nascimento et al., 2006), antimicrobial (Jardim, Jham, Dhingra, & Freire, 2008; Liu, Liu, Zhang, Li, & Cheng, 2013; Pandey, Singh, Palni, & Tripathi, 2013), antiparasitic (Guerra, Torres, & Martínez, 2001; Kiuchi et al., 2002; Monzote et al., 2004), anti‐inflammatory, and antinociceptive (Trivellato‐Grassi et al., 2013) effects.

**Figure 1:**
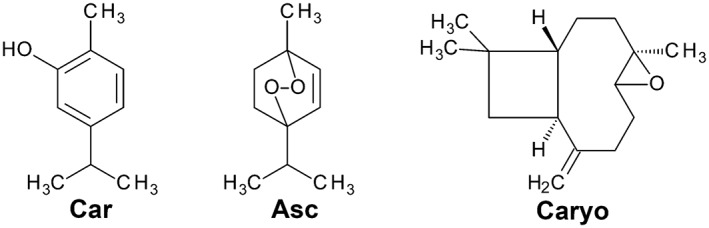
Chemical structure of main compounds of essential oil from Chenopodium ambrosioides L.: carvacrol (Car, 62%), ascaridole (Asc, 22%), and caryophyllene oxide (Caryo, 5%) according to Monzote et al. (2006)

In a series of previous studies, we observed the antileishmanial potential of essential oil (EO) from C. ambrosioides L. in different in vitro and in vivo models (Monzote et al., 2006; Monzote, Garcia, et al., 2014). In parallel, in the chromatogram obtained by gas chromatography/mass spectrometry, we identified three major compounds of the EO (Figure 1), namely, carvacrol (Car) with 62%, ascaridole (Asc) with 22%, and caryophyllene oxide (Caryo) with 5% of total content (Monzote et al., 2006). These three compounds were also identified in EO of Chinese C. ambrosioides L., however, in different percentages (Chu, Hu, & Liu, 2011). In addition to these volatile components, this EO contains also nonvolatile (solvent extractable) pharmacologically active compounds (Shah & Khan, 2017). Car, Caryo, and Asc showed antileishmanial activity, although they were less selective for *Leishmania* in comparison with mammalian host cells than EO for *Leishmania* compared with effects on mammalian host cells (Monzote et al., 2006; Monzote, Garcia, et al., 2014). Asc, which is also present in tea tree oil, demonstrated a skin‐sensitizing effect in mammals (Chittiboyina, Avonto, & Khan, 2016; Krutz et al., 2015). By the use of iron chelators, it was shown that activation of the endoperoxide Asc in EO by iron is essential for its antiparasitic actions. Nevertheless, differences in the activity profile of Asc and EO have been observed in the system of macrophages/*Leishmania*.

In addition, it has been demonstrated that one possible toxicological mechanism behind the actions of EO and its major components against mammalian cells is related to mitochondrial dysfunction (Monzote, Stamberg, Staniek, & Gille, 2009). In *Leishmania*, there have been indications that EO and its major compounds also influence mitochondrial functions (Monzote, Garcia, et al., 2014), but specific mechanisms and targets have not been identified so far.

Herein, we study the effects of EO's main compounds (Asc, Car, and Caryo) on electron transport chain (ETC) complexes I–III in different models (yeast, *Leishmania*, and mammals) at the molecular level. Short‐term effects as well as long‐term effects of Asc have been investigated with the objective to elucidate the role of mitochondrial effects in the EO actions in *Leishmania*.

## 2 METHODS

### 2.1 Chemicals

Diethylenetriaminepentaacetic acid (DTPA, sodium salt), dimethyl sulfoxide (DMSO), ethylenediamine tetraacetic acid (EDTA), glucose, HCl, KCN, KH_2_PO_4_, Na_2_HPO_4_, NaH_2_PO_4_, NaCl, NaN_3_, sodium dodecyl sulphate, succinate, sucrose, and tris(hydroxymethyl)aminomethane (Tris) were obtained from Merck (Germany). Bovine serum albumin (BSA), decylubiquinone (dUQ), 2,6‐dichlorophenol–indophenol (DCPIP), dithiothreitol (DTT), cytochrome *c* ^3+^ (cyt *c* ^3+^), glutathione, hemin, hydroxyapatite, phenylmethylsulfonyl fluoride, penicillin–streptomycin solution, reduced nicotinamide–adenine dinucleotide (NADH), resazurin, sorbitol, Schneider's medium, brain heart infusion (BHI) medium, oligomycin (Oligo), antimycin A (AA), carbonyl cyanide‐*m*‐chlorophenylhydrazone (CCCP), paraffin oil, and Triton X‐100 were purchased from Sigma (USA). Triethanolamine hydrochloride was from Fluka (Switzerland), whereas Desferal (desferrioxamine mesylate [DFO]) was from Novartis Pharma (Germany), and 1‐hydroxy‐3‐methoxycarbonyl‐2,2,5,5‐tetramethylpyrrolidine‐HCl (CMH) was from Noxygen (Germany). Idebenone and zymolyase were from Takeda and Seikagaku Corporation (Japan), respectively. Yeast extract powder was supplied by Amresco (USA). 5‐Chloromethylfluorescein diacetate (CMFDA) was purchased from Abcam (USA).

### 2.2 EO from C. ambrosioides L. and its main compounds

In this study, we used an aliquot of the original sample (stored at −20 °C) for which the type of collection, extraction of EO, and chemical characterization were described previously (Monzote et al., 2006). Briefly, aerial part of C. ambrosioides L. plant in flowering stage was collected in July, and voucher specimen number (ROIG4639) was assigned at the Experimental Station of Medicinal Plants “Dr Juan Tomás Roig,” Cuba. The EO was extracted from fresh material by hydrodistillation in a Clevenger apparatus over 4 hr to yield approximately 1% oil (Monzote et al., 2006). Asc was obtained by chemical synthesis by addition of singlet oxygen to α‐terpinene using rose bengal as a photosensitizer (Monzote et al., 2009). Structure and stability of the product was studied by nuclear magnetic resonance (NMR), and a purity of around 95% was determined. ^1^H NMR (400.13 MHz, CDCl_3_): δ [ppm] 6.49 (d, *J* = 8.6 Hz, 1H, H‐3), 6.31 (d, *J* = 8.6 Hz, 1H, H‐2), 2.03 (m, 2H, H‐5a, H‐6a), 1.92 (sept, *J* = 7.0 Hz, 1H, H‐7), 1.51 (m, 2H, H‐5b, H‐6b), 1.37 (s, 3H, 4‐Me), 1.00 (d, *J* = 7.0 Hz, 6H, H‐8). δ = 7.29 ppm = solvent (CDCl_3_) and δ = 1.60 ppm = H_2_O. ^13^C NMR (100.61 MHz, CDCl_3_): δ [ppm] 17.12 (isopropyl CH_3_), 17.20 (isopropyl CH_3_), 21.37 (4‐Me), 25.58 (C‐6), 29.51 (C‐5), 32.11 (isopropyl C), 74.32 (C‐4), 79.76 (C‐1), 133.03 (C‐3), 136.37 (C‐2; assignments according to Cavalli, Tomi, Bernardini, & Casanova, 2004). The accordance of this NMR data with our previously published data (Monzote et al., 2009) confirms the stability and purity of Asc. Car and Caryo were from Sigma (USA), with a purity of >98% and >95%, respectively. All products were diluted with DMSO.

### 2.3 Parasite culture

*Leishmania tarentolae* promastigotes (LtP) strain P10 from Jena Bioscience (Germany) was used. Parasites were cultured at 26 °C either in yeast extract medium (YEM; 20.7 g/L yeast extract powder, 0.2 g/L KH_2_PO_4_, 1.2 g/L K_2_HPO_4_, and 2.9 g/L glucose) or in BHI medium (37 g/L) supplemented with 5 mg/L hemin and 50,000 U/L penicillin—50 mg/L streptomycin.

### 2.4 Preparation of mitochondrial fractions

#### 2.4.1 Isolation of mitochondrial fractions from LtP

LtP culture (2,700 ml) was centrifuged at 478 *g* over 10 min at 4 °C (Sorvall RC26 Plus, USA). The supernatant was discarded, and the cell pellet was resuspended in buffer (10 mM Tris–HCl, 0.3 M sucrose, 0.2 mM EDTA, and 0.2% BSA, pH 7.4). Following two repeated washes (478 *g*, 10 min at 4 °C), the resulting cell pellet was incubated in lysis buffer (5 mM Tris–HCl, pH 7.4) for 10 min at 25 °C and subsequently homogenized in a Dounce homogenizer. Cell debris was removed by centrifugation (1,005 *g*, 10 min, 4 °C). The supernatant was again centrifuged (13,176 *g*, 20 min, 4 °C) to sediment the mitochondrial fraction (LtP‐Mit). The mitochondria were resuspended in 1 ml of buffer (250 mM sucrose, 50 mM KH_2_PO_4_, and 0.2 mM EDTA, pH 7.2) and stored in liquid nitrogen until use.

#### 2.4.2 Isolation of submitochondrial particles from bovine heart

Bovine heart submitochondrial particles (BH‐SMP) were obtained from bovine heart mitochondria by sonication (Nohl & Hegner, 1978) and stored in liquid nitrogen until use.

#### 2.4.3 Isolation of SMP from yeast

Yeast mitochondria were prepared from Saccharomyces cerevisiae yeast (ScY) strain DBY 747 (Daum, Bohni, & Schatz, 1982). Cells were harvested by centrifugation (1,464 *g*, 5 min, 20 °C), and the pellet was resuspended in buffer I (10 mM Tris and 10 mM DTT, pH 9.4). Following 15 min of incubation at 37 °C, cells were centrifuged (1,464 *g*, 5 min, 20 °C) and resuspended in buffer II (1.2 M sorbitol and 20 mM KH_2_PO_4_, pH 7.4). Finally, after a repeated centrifugation (1,464 *g*, 5 min, 20 °C), the weight of the cell pellets was determined. To prepare spheroblasts, pellets were suspended in buffer II complemented with 2 mg zymolyase/g yeast cells. After incubation for 45 min at 28 °C, spheroblasts were collected by centrifugation (1,464 *g*, 5 min, 20 °C), resuspended in 30 ml of buffer II, sedimented again (5 min at 1,464 *g* and 20 °C), and homogenized in 30 ml of buffer III (600 mM sorbitol and 20 mM Tris, pH 7.4) using a Wheaton Dounce tissue grinder. Cells and cell debris were removed by two centrifugations (1,464 *g*, 5 min, 4 °C). Mitochondria were finally collected from the supernatant by centrifugation (11,952 *g*, 10 min, 4 °C) and used to prepare ScY submitochondrial particles (ScY‐SMP). Mitochondrial pellets were suspended in 5 ml of buffer I (without DTT) and diluted to 25 ml with 10 mM Tris (pH 7.5). The suspension was kept on ice for 20 min followed by a centrifugation (39,500 *g*, 10 min, 4 °C). The pellet was resuspended in 20 ml of sucrose buffer (250 mM sucrose and 10 mM Tris, pH 7.4) and sonicated 18 times for 20 s (Branson sonifier at maximum intensity) with interruptions of 10 s for heat dissipation. Subsequently, the suspension was centrifuged (5,400 *g*, 10 min, 4 °C) to remove mitochondria. ScY‐SMP were sedimented from the supernatant by centrifugation (195,000 *g*, 60 min, 4 °C). The obtained ScY‐SMP pellet was homogenized in 1.5 ml of buffer (250 mM sucrose, 0.2 mM EDTA, and 50 mM KH_2_PO_4_, pH 7.2) and stored in liquid nitrogen.

#### 2.4.4 Isolation of cytochrome *bc*_1_ complex

Cytochrome *bc*_1_ complex was obtained from ScY‐SMP and BH‐SMP following published methodology (Geier, Schägger, Brandt, Colson, & von Jagow, 1992) with minor modifications as described below. SMP were resuspended in 1.1% Triton X‐100 and 200 mM phenylmethylsulfonyl fluoride, pH 7.2, and centrifuged for 1 hr at 100,000 *g*. The *bc*_1_ complex in the sediment was solubilized with 2.2% Triton X‐100, 600 mM NaCl, 10 mM KH_2_PO_4_, and 10 mM EDTA, pH 7.2. After centrifugation at 100,000 *g* for 1 hr, the supernatant was mixed with 50 ml of hydroxyapatite, equilibrated with 0.5% Triton X‐100, 250 mM NaCl, and 100 mM NaHPO_4_, pH 7.2. After washing the hydroxyapatite with 50 ml of equilibration buffer (0.05% Triton X‐100, 100 mM NaHPO_4_, and 250 mM NaCl), the *bc*_1_ complexes from ScY‐SMP and BH‐SMP (ScY‐*bc*_1_ and BH‐*bc*_1_, respectively) were eluted from hydroxyapatite with 250 mM KH_2_PO_4_ and 0.25% Triton X‐100, pH 7.2, and stored in liquid nitrogen.

### 2.5 Determination of protein and cell concentrations

The protein concentration of mitochondrial preparations was determined by the Biuret method (Lowry, Rosebrough, Farr, & Randall, 1951). The number of LtP was determined by optical density at 600 nm (HITACHI U‐1100 Spectrophotometer, Japan). The cell broth was diluted 1:10 with culture medium and measured against a blank of culture medium. The cell number was calculated using the formula C_Cell_ (10^6^/ml) = OD_600nm_ * 0.969 * 124 (Fritsche, Sitz, Weiland, Breitling, & Pohl, 2007). Two replicates of each culture were performed.

### 2.6 Viability of LtP treated with EO main components

LtP (200 μl, 2 × 10^6^ parasites/ml) in YEM/phosphate‐buffered saline (PBS; 1:1 vol/vol, including antibiotics and 6 μM hemin) were distributed in 96‐well plates (nontreated plates). Compound stocks (in DMSO, max. 2% final concentration) were added, and five 1:3 serial dilutions were performed. Control rows with YEM/PBS (no activity) and with untreated LtP (vehicle, DMSO; 100% activity) were loaded, and the plate was incubated at 26 °C for 48 hr. Then resazurin (50 μl in PBS) was added (20 μM final concentration), and after 4 hr of incubation at 26 °C, the fluorescence of resazurin was measured at 560 nm excitation and 590 nm emission using a plate reader (PerkinElmer Enspire, Germany). Each compound was tested in triplicate.

### 2.7 Activity of EO compounds on ETC complexes in LtP‐Mit and BH‐SMP

#### 2.7.1 Influence on NADH:ubiquinone oxidoreductase (complex I) and succinate:ubiquinone oxidoreductase (complex II) activities

NADH:ubiquinone oxidoreductase (complex I) and succinate:ubiquinone oxidoreductase (complex II) activities were determined in 96 well‐plates using DCPIP as the electron acceptor. In each well, 200 μl of premix containing buffer (250 mM sucrose, 20 mM triethanolamine, and 1 mM EDTA, pH 7.4), DCPIP (60 μM), BSA (3.5 mg/ml for complex I and 1 mg/ml for complex II), idebenone (50 μM), and LtP‐Mit (complex I 0.17 μg/ml protein and complex II: 51 μg/ml protein) or BH‐SMP (complexes I and II: 8 μg/ml protein) were added. In addition, in the first wells, 97 μl of premix and 3 μl of EO compounds were added. Then six serial 1:3 dilutions were carried out transferring 100 μl in each step. The reaction was started by adding 50 μl of start‐mix per well, giving final concentrations of KCN (1 mM), for complex I:NADH (300 μM) or complex II:succinate (4 mM). After start of the reaction, images of the plates were recorded with a Canon EOS 300D camera (PLReader software, red–green channel) each 10 min for LtP‐Mit and 3 min for BH‐SMP (20 absorbance measurements). For each EO compound, percentage of inhibition was obtained with respect to controls (set to 100%) treated with maximum volume of vehicle (DMSO) introduced by test compound stocks.

#### 2.7.2 Influence on ubiquinol:cytochrome *c* oxidoreductase activity

To measure the ubiquinol:cyt *c* ^3+^ oxidoreductase (complex III) activity, the reduction of 100 μM cyt *c* ^3+^ at 550 nm using 540 nm as reference was monitored in the presence of the artificial substrate decylubiquinol (dUQH_2_, 75 μM), which was prepared from dUQ by reduction (Müllebner et al., 2010). The dUQH_2_:cyt *c* ^3+^ oxidoreductase activities of 40 μg protein/ml of LtP‐Mit or 3.2 μg protein/ml of BH‐SMP were measured in 1 ml of buffer containing 250 mM sucrose, 50 mM KH_2_PO_4_, 0.2 mM EDTA (pH 7.2), 2 mM KCN, and 4 mM NaN_3_. Respective EO compounds were added 50 s after starting the time scan, and the reaction was started after 100 s with dUQH_2_ and was monitored for additional 150 s. The activity of noninhibited dUQH_2_:cyt *c* ^3+^ oxidoreductase activity was measured in the presence of the vehicle for the respective inhibitor (DMSO). All inhibitor concentrations were tested in triplicate. The reduction rates for cyt *c* ^3+^ were calculated from the time trace of the absorption difference at 550 − 540 nm (ε_550–540 nm_ = 19 mmol^−1^ L cm^−1^). Reduction rates in the presence of DMSO (maximum amount that was introduced by test compound stocks) were set to 100%, and the remaining activities in the presence of EO compounds were expressed in %. Three replicates were measured for each concentration.

### 2.8 Influence of EO compounds on isolated *bc*_1_ complex

To determine the influence of EO compounds on purified *bc*_1_ complex, the dUQH_2_:cyt *c* ^3+^ oxidoreductase activities were measured as described. In this case, concentrations of 21.3 μg/ml of ScY‐*bc*_1_ or 1.6 μg/ml of BH‐*bc*_1_ were used.

### 2.9 Oxygen consumption of LtP

#### 2.9.1 Clark electrode

Direct inhibitors of mitochondrial ETC instantaneously influence mitochondrial oxygen consumption. To assay the effect of EO compounds on oxygen consumption of LtP, a Clark‐type oxygen electrode (Hansatech, Germany) and software MCREC were used. LtP at approximately 10^8^ cells/ml in YEM (25 °C) were added and treated with increasing concentrations of EO compounds between 10 and 200 μM and for EO 5.6–89.6 μg/ml. Each concentration was assayed in quadruplicates, and the results were expressed as percentage of oxygen consumption in comparison with the untreated control LtP. The highest concentration of the vehicle (1% DMSO) caused only 2% inhibition. The uncoupling effect in short‐term and long‐term (0, 6, and 24 hr) incubations with Asc (200 μM) was studied in Oligo‐inhibited (5 μM) and uncoupler‐stimulated (0.5 μM CCCP) LtP in Schneider's medium supplemented with 6 μM hemin. Four replicates were performed for each concentration.

#### 2.9.2 OxoPlates

U‐shaped 96‐well OxoPlates (OP96U PreSens, Germany) with integrated fluorescence oxygen sensors were used for parallel LtP experiments. Oxygen concentrations were measured using a PerkinElmer Enspire fluorescence plate reader using excitation wavelength 540 nm and two emission wavelengths (reference dye 590 nm, I_Ref_, and O_2_‐sensing dye 650 nm, I_Ind_). The oxygen concentration (μM O_2_) was calculated according to manufacturer's instructions as described previously (Monzote et al., 2016). Measurements with LtP were performed in air‐saturated BHI medium. After calibration of the OxoPlates, they were loaded with 200 μl medium (medium controls for drift corrections), 50 μl medium in wells for untreated control cells, or 50 μl medium with EO compounds (Car, Asc, and Caryo) or uncouplers of mitochondrial respiration in wells for treated cells. Immediately before the measurement, 150 μl of well‐aerated cell suspensions (13 × 10^7^ LtP per ml) was added to the respective wells. Finally, on the top of each well, 50 μl of paraffin oil was layered. Two minutes after mixing, the fluorescence measurements at 27 °C were started, and eight measurements at 5‐min intervals were performed. From the linear part of the O_2_ decay, the slopes were calculated and corrected for the medium drift for further statistic evaluation. All measurements were performed at least in triplicate. For bioenergetic characterization, the ATP synthase inhibitor Oligo (5 μM) and as positive control CCCP (0.7–5 μM) were used. Respiratory control ratio (RCR) was calculated from respiration rate in the presence of Oligo and compounds/uncoupler divided by the rate in the presence of Oligo.

### 2.10 Measurement of superoxide radicals

Detection of superoxide radicals was performed using CMH as reaction partner and measuring the formed stable nitroxyl radicals by electron spin resonance (ESR) spectroscopy. Measurements were performed in PBS (136 mM NaCl, 1.15 mM KH_2_PO_4_, 14 mM Na_2_HPO_4_, and 2.7 mM KCl, pH 7.4) containing 15 mM glucose, 400 μM CMH, and 100 μM DFO (desferrioxamine mesylate) and 25 μM DTPA. Usually, these assays are performed in the presence of iron chelators (DFO and DTPA) to prevent CMH interaction with free Fe^3+^ leading to CM^●^ unrelated to superoxide radicals. Before the measurements, LtP suspensions were washed twice with PBS to remove the medium. Typical experiments contained 5 × 10^8^ LtP cells/ml. Stock solutions of EO compounds dissolved in DMSO were added. The influence of Asc (6–700 μM) on the CMH oxidation rate by LtP was assessed immediately and after prolonged incubation time (1 hr between Asc and CMH addition). The influence of iron‐dependent Asc activation in LtP on CMH oxidation was studied in the absence of iron chelators DFO and DTPA. Prior to the ESR measurement, 400 μM CMH was added. Afterwards, 17 μl of suspension was aspirated in gas‐permeable Teflon tubes (ID 0.7 mm). This capillary tube was placed in the resonator of the ESR instrument (Bruker EMX, split ring MD5, Germany), and 10 sequential measurements were performed. The following instrument settings were used: microwave frequency 9.682 GHz, modulation frequency 100 kHz, modulation amplitude 1 G, time constant 0.082 s, center field 3,446 G, scan rate 71 G/min, sweep width 100 G, scan time 84 s, and attenuation CMH: 7.96 × 10^3^. From the ESR spectra, the middle peak intensity was retrieved, and concentrations of oxidized CMH were obtained by comparison with a standard curve prepared from 3‐carboxy‐proxyl solutions with defined concentrations. Four replicate measurements were performed for each condition.

### 2.11 Measurement of low molecular thiols in LtP

LtP were centrifuged twice (1,800 *g*, 10 min, 20 °C), the cell pellets were resuspended in PBS/glucose (15 mM), and the cell number was adjusted to 1 × 10^7^/ml. Aliquots (250 μl) were added to 96‐well plates (black, nontreated plates), along with compound stocks (keeping a maximum of 2% DMSO). Following a 5‐hr incubation at 26 °C, plates were centrifuged (1,800 *g*, 10 min, 20 °C), and 200 μl of supernatant was removed. To the remaining cell suspension, 100 μl of CMFDA in PBS was added giving a final concentration of 5 μM. After gentle shaking, the cells were incubated for 15 min at 37 °C in the dark. The fluorescence of the methyl fluorescein (MF)‐thiol adduct (Sarkar, Mandal, Singh, Sundar, & Chatterjee, 2009) was measured at 15, 30, 45, and 60 min at 485 nm (excitation wavelength) and 530 nm (emission wavelength) using a plate reader (PerkinElmer Enspire, Germany). The OD_600 nm_ was determined for cell count, and the measured fluorescence intensity was normalized to the cell number. Three replicate measurements were performed for each condition.

### 2.12 Statistical analysis

The median of inhibitory concentration (IC_50_) value was determined from nonlinear concentration–response curves using Origin^®^ Program Version 6.1 and expressed as the mean ± standard deviation. Statistically significant differences of *p* < .05 were identified using Student's *t* test.

## 3 RESULTS

### 3.1 Antileishmanial activity of EO components

Viability assays for LtP resulted in IC_50_ values for Asc of 24.5 ± 3.0 μM, Car of 11.6 ± 3.4 μM, and Caryo of 36.0 ± 17.6 μM (*n* = 3). For EO, an IC_50_ value of 19.1 ± 6.1 μg/ml was determined. This indicates that all major EO components possibly contribute to the antileishmanial action of EO.

### 3.2 Inhibition of oxygen consumption

**Figure 2:**
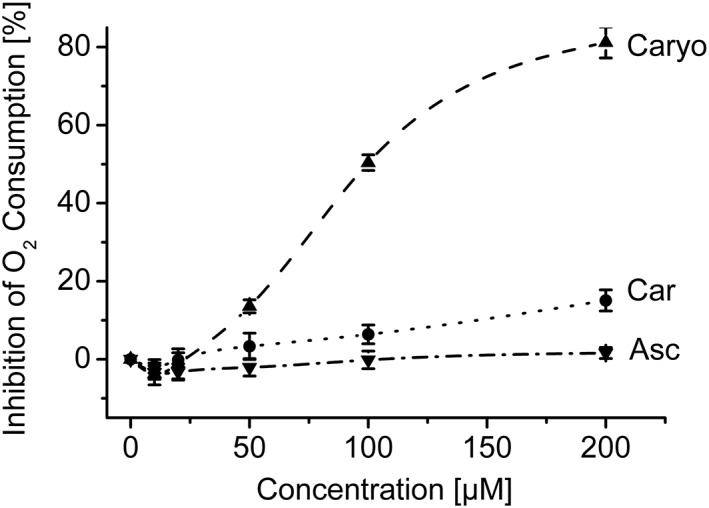
Influence of major components of essential oil from Chenopodium ambrosioides L. on the cellular oxygen consumption of *Leishmania tarentolae* promastigotes (LtP). Oxygen consumption of LtP (72–100 × 10^6^ cells/ml) was assessed by a Clark‐type electrode in air‐saturated medium containing 14.6 mM glucose. Increasing concentrations of compounds were added subsequently using DMSO as vehicle. At 1% DMSO (highest final concentration), O_2_ consumption of LtP was inhibited by 1.74 ± 9.46%. Data are means ± standard deviation of four independent experiments. Asc = Ascaridole; Car = carvacrol; Caryo = caryophyllene oxide

EO inhibited the leishmanial oxygen consumption in concentrations above 20 μg/ml (IC_50_ being 66.6 ± 6.4 μg/ml). The assayed components Asc, Car, and Caryo are sufficiently lipophilic to be immediately taken up by *Leishmania*; it suggests that they can reach leishmanial mitochondria within minutes; accordingly, we examined their impact on LtP mitochondria. Therefore, mitochondrial function was evaluated by measurement of oxygen consumption of LtP in the presence of EO components (Figure 2). Car and Asc failed to strongly inhibit oxygen consumption, whereas Caryo strongly inhibited LtP oxygen consumption, IC_50_ being 98.0 ± 2.0 μM. This effect could contribute to the inhibition by EO because it contains about 5% Caryo.

### 3.3 Inhibition of mitochondrial complexes

In general, no strong inhibition was observed for complexes I and II (Table 1). However, complex III inhibition of LtP‐Mit by Caryo confirmed its interference at this site. In contrast, for BH‐SMP, the inhibitory effect of Caryo was weaker. Asc and Car showed no strong inhibition in the studied concentration ranges suggesting that they have no specific targets in the ETC of *Leishmania* and mammals (Table 1).

**Table 1:** Influence of major EO components on the LtP‐Mit in comparison with BH‐SMP on mitochondrial activities of complexes I–III

Compound	IC_50_ (μM; % of inhibition at highest concentration tested)					
	Complex I		Complex II		Complex III	
	LtP‐Mit	BH‐SMP	LtP‐Mit	BH‐SMP	LtP‐Mit	BH‐SMP
Asc	>100 (1.1)	>100 (5.1)	>100 (17.6)	>100 (21.2)	131.3 ± 3.5	>100 (17.7)
Car	>100 (16.4)	>100 (13.3)	>100 (8.8)	>100 (21.5)	150.1 ± 2.3	>100 (22.8)
Caryo	>100 (7.2)	>100 (39.7)	>100 (12.3)	>100 (16.3)	54.8 ± 4.0	>100 (10.0)

*Note*. IC_50_ > value: In these cases, the IC_50_ was not determined because only an inhibition <50% was observed at highest concentration. In parentheses, % of inhibition at highest concentration tested. Results were expressed as mean ± SD or as percentage of three independent experiments. Asc = ascaridole; BH‐SMP = submitochondrial particles from bovine heart; Car = carvacrol; Caryo = caryophyllene oxide; EO = essential oil; LtP‐Mit = mitochondrial crude fraction of *Leishmania tarentolae*; SD = standard deviation.

In a next step, we extended our experiments to isolated ScY‐*bc*_1_ and BH‐*bc*_1_ (Table 2). Among major compounds of EO, Caryo exhibited the strongest inhibition, whereas Asc and Car were less effective (Table 2), suggesting that the immediate effects of EO major components on mitochondria in all studied species are mediated by Caryo and not by the major antileishmanially active compound Asc.

**Table 2:** Immediate inhibitory activity of main compounds of EO from Chenopodium ambrosioides L. on ScY‐*bc*_1_ and BH‐*bc*_1_

Compound	IC_50_ ± SD (μM)	
	ScY‐*bc*_1_	BH‐*bc*_1_
Asc	675 ± 6	381 ± 9
Car	941 ± 41	421 ± 4
Caryo	179 ± 1	197 ± 1

Results were expressed as median inhibitory concentration (IC_50_) ± SD of five independent experiments. Asc = ascaridole; BH‐*bc*_1_ = cytochrome *bc*_1_ complex purified from Bos taurus; Car = carvacrol; Caryo = caryophyllene oxide; EO = essential oil; ScY‐*bc*_1_ = cytochrome *bc*_1_ complex purified from Saccharomyces cerevisiae; SD = standard deviation.

### 3.4 Influence on cellular superoxide radical production

**Figure 3:**
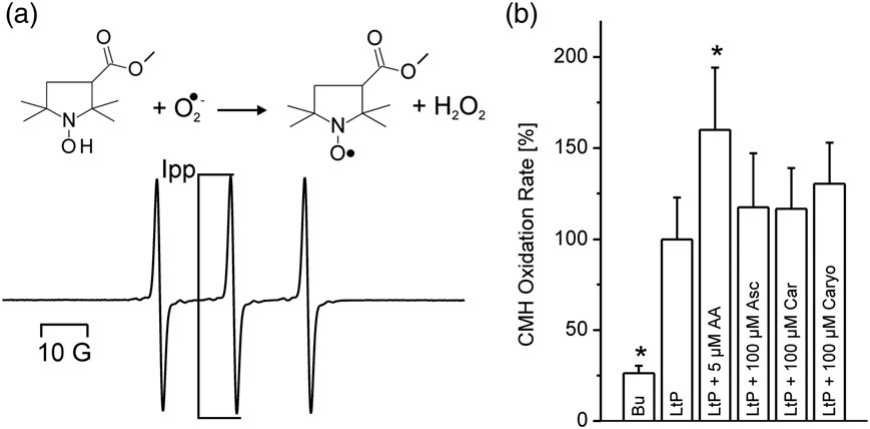
Detection of superoxide radicals in *Leishmania tarentolae* promastigotes (LtP) and the influence of major compounds of essential oil from Chenopodium ambrosioides L. Superoxide radicals in LtP converted the cyclic hydroxylamine CMH to a stable nitroxyl radical shown in (a). The intensity of the middle peak (Ipp) is proportional to the amount of superoxide formed. (b) The influence of major components of essential oil (100 μM) and antimycin A (AA, 5 μM) on the radical formation in LtP. Buffer (Bu) indicates samples without LtP. The reaction with 400 μM CMH was performed in suspensions containing 5 × 10^8^ LtP/ml in phosphate‐buffered saline with 15 mM glucose, 100 μM DFO, and 25 μM DTPA. Data represent mean ± standard deviation of quadruplicate experiments. ^*^Significant differences versus LtP on the level *p* < .1. Asc = Ascaridole; Car = carvacrol; Caryo = caryophyllene oxide

In a next experiment, we studied superoxide radical formation in LtP by an ESR method using cyclic hydroxyl amine (CMH), which is converted upon one‐electron transfer reaction with superoxide radicals to a stable nitroxyl radical (CM^●^, Figure 3a). As can be seen (Figure 3b), LtP significantly triggered CMH oxidation in comparison with buffer. Also, the positive control with AA, a well‐known trigger of mitochondrial superoxide formation, showed an increase of CM^●^ formation in LtP. Asc, Car, and Caryo only slightly increased CM^●^ formation. At first view, it appears puzzling that Asc, which is known to trigger formation of carbon‐centered radicals (Geroldinger et al., 2017), does not trigger superoxide formation in this assay.

### 3.5 Activation of Asc and cellular superoxide radical production

**Figure 4:**
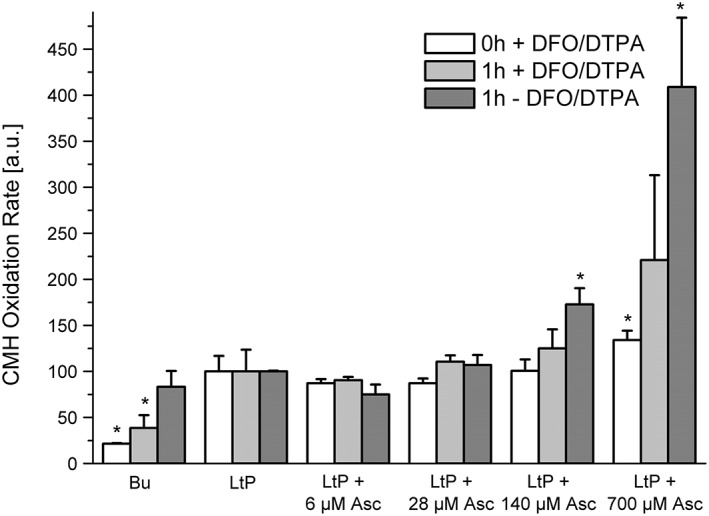
Formation of superoxide radicals in *Leishmania tarentolae* promastigotes (LtP) triggered by ascaridole (Asc) increased with Asc concentration, incubation time, and iron availability. Superoxide radical formation was assessed by reaction of 400 μM CMH in suspensions containing 5 × 10^8^ LtP/ml in phosphate‐buffered saline with 15 mM glucose and 100 μM DFO and 25 μM DTPA (white bars). Immediate effects (0‐hr incubation) of Asc in LtP in the presence of chelators (DFO/DTPA). After 1‐hr incubation (light grey) and after 1‐hr incubation in the absence of iron chelators (dark grey). Data represent mean ± standard deviation of quadruplicate experiments. ^*^Significant differences versus LtP on the level *p* < .05. Bu = buffer controls without LtP

The dose‐dependent increase of CMH oxidation triggered by Asc becomes increasingly effective upon prolonged incubation and in the absence of iron chelators (Figure 4) suggesting that superoxide radical formation by Asc does not occur immediately and is enhanced by the availability of iron allowing for Asc activation.

### 3.6 Influence on mitochondrial coupling

**Figure 5:**
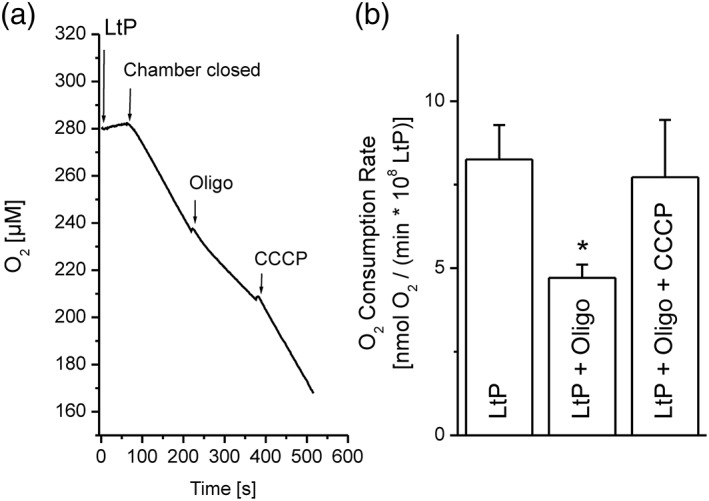
Mitochondrial coupling in *Leishmania tarentolae* promastigotes (LtP). (a) Typical experiment to assess mitochondrial coupling in LtP. LtP cells (110 ± 13 × 10^6^/ml) in Schneider's medium supplemented with 6 μM hemin were equilibrated with air within the reaction chamber of a Clark‐type electrode. After closing the chamber, the basal cellular O_2_ consumption was measured. By addition of 5 μM oligomycin (Oligo), an inhibitor of ATP synthase, oxygen consumption coupled to ATP production was blocked. Finally, the uncoupler CCCP (0.5 μM) was added to obtain the capacity of mitochondrial electron transport in LtP at non‐coupled respiration. (b) O_2_ consumption rates obtained from the respective slopes in the left graph. Data represent mean ± standard deviation of four independent experiments. ^*^Significant differences versus LtP on the level *p* < .05

Although Asc is not a direct inhibitor of ETC in LtP (Figure 2), a decrease in the membrane potential of *Leishmania amazonensis* promastigotes (LaP), as assessed by JC‐1, was observed after 72 hr of incubation (Monzote, Garcia, et al., 2014), and Asc was shown to produce radicals in LtP (Geroldinger et al., 2017). Accordingly, the long‐term effects of Asc on mitochondrial coupling in LtP were examined by measuring the respiration of LtP with a Clark‐type electrode. Upon addition of Oligo (inhibitor of ATP synthase), oxygen consumption stimulated by ATP production was blocked (Figure 5), and inclusion of the uncoupler CCCP yielded the maximally uncoupled respiration. The quotient of both rates, the RCR, is an indication of the ability of LtP to respond to increased ATP demands due to stress conditions. Therefore, a high RCR is indicative of a healthy cell, and a decreased RCR reflects an impaired stress response.

**Figure 6:**
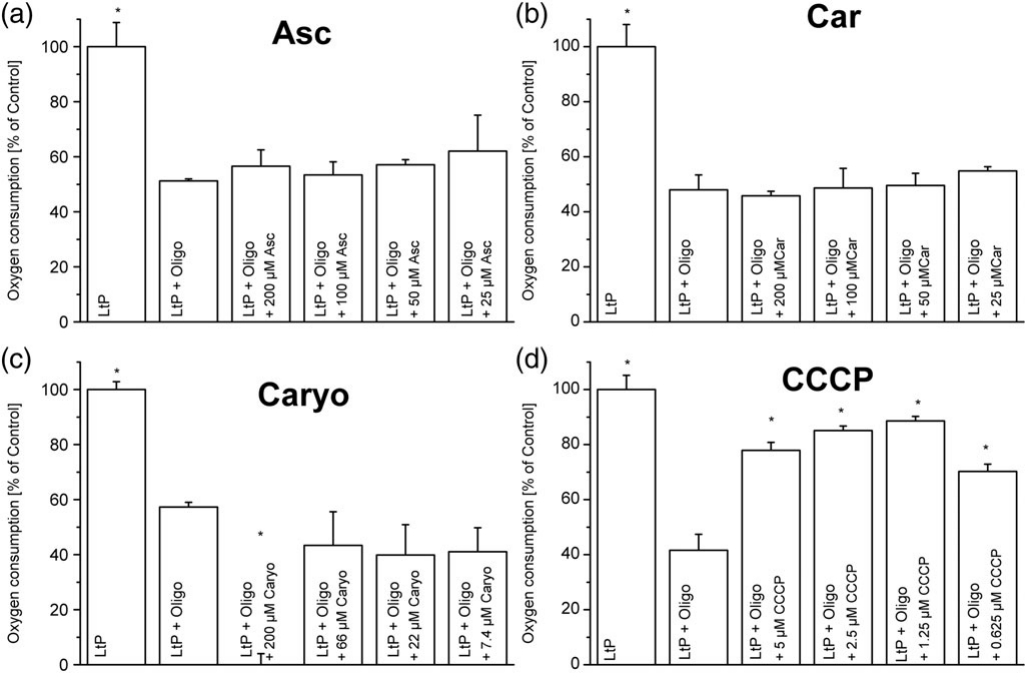
Mitochondrial uncoupling in *Leishmania tarentolae* promastigotes (LtP) in the presence of major components of essential oil from Chenopodium ambrosioides L. Uncoupling was assessed by stimulation of oxygen consumption in the presence of the ATP synthase inhibitor oligomycin (Oligo). LtP cells (1 × 10^8^/ml) in brain heart infusion medium were supplemented with Oligo (5 μM) and decreasing concentrations of ascaridole (Asc), carvacrol (Car), caryophyllene oxide (Caryo), and the uncoupler CCCP. Oxygen consumption was assessed in 96‐well OxoPlates for 40 min at 26 °C. Oxygen consumption rates were normalized to the respiration of non‐inhibited *Leishmania* (LtP = 100%). Data represent mean ± standard deviation of 3–4 experiments. ^*^Significant differences versus LtP + Oligo on the level *p* < .05

**Figure 7:**
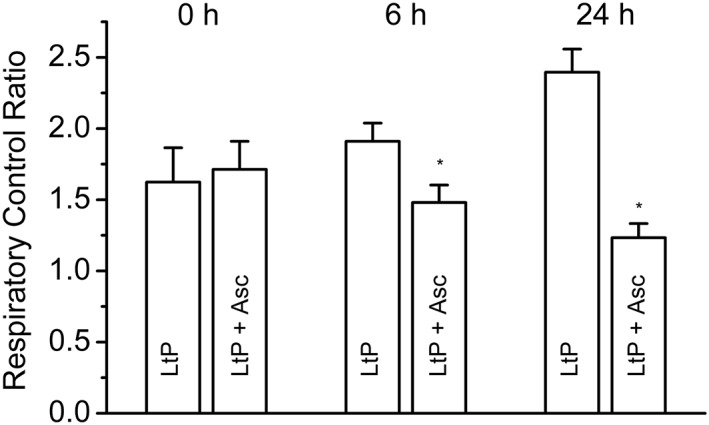
Effects of ascaridole (Asc) on mitochondrial coupling in *Leishmania tarentolae* promastigotes (LtP) upon prolonged incubation. Different cell batches with 1 × 10^8^ LtP/ml in Schneider's medium plus 6 μM hemin were incubated in culture tubes either with DMSO (vehicle for Asc; LtP) or with 200 μM Asc (LtP + Asc). From these culture stocks, aliquots were taken for O_2_ consumption measurements at 0, 6, and 24 hr. Mean cell counts during measurements were adjusted with medium to approximately 1–2 × 10^8^ LtP/ml. Respiratory control ratios were calculated as the ratios of O_2_ consumption rates in the presence of 5 μM oligomycin plus 0.5 μM CCCP to oligomycin‐inhibited O_2_ consumption rates as shown in Figure 5. Data represent mean ± standard deviation of four independent experiments. ^*^Significant differences versus LtP on the level *p* < .05

In a first experimental series, we tested the immediate uncoupling effect of EO major components in 96‐well OxoPlates. Neither Car nor Asc (Figure 6a,b) showed an uncoupling effect under these conditions. Caryo at the highest concentration (200 μM) totally inhibited the oxygen consumption on top of the inhibition by Oligo, confirming the interference of Caryo in the leishmanial ETC (Figure 6c). In contrast, the positive control with the uncoupler CCCP showed a clear increase of Oligo‐inhibited respiration in LtP (Figure 6d), demonstrating the functionality of this assay. As expected, Asc showed only little effects on mitochondrial coupling in LtP immediately after addition (Figure 7). However, upon prolonged incubation with Asc, the RCR declined with respect to vehicle‐treated LtP suggesting an impaired cellular energy metabolism.

### 3.7 Influence on low molecular thiols

**Figure 8:**
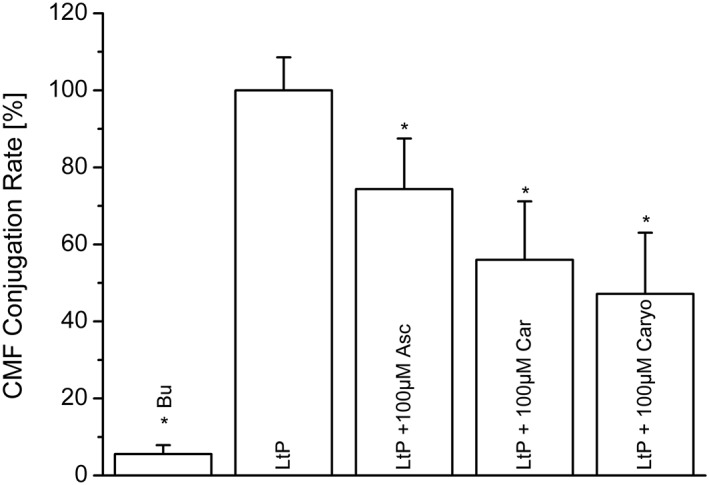
Influence of major components of essential oil from Chenopodium ambrosioides L. on the low molecular thiol status of *Leishmania tarentolae* promastigotes (LtP) after 5 hr incubation at 26 °C in phosphate‐buffered saline/glucose medium. Low molecular thiol status was assessed by measuring the rate of fluorescence evolution over 1 hr from the conjugation of CMF (arising from 5 μM CMFDA) to low molecular thiols in LtP (1 × 10^7^ cells/ml) in phosphate‐buffered saline/glucose. Results represent mean ± standard deviation of three experiments. ^*^Significant differences versus LtP on the level *p* < .05. Asc = Ascaridole; Bu = buffer; Car = carvacrol; Caryo = caryophyllene oxide

As an indicator of oxidative stress, the status of low molecular thiols was assessed by the CMFDA method (Sarkar et al., 2009) wherein CMFDA is intracellularly deacetylated to CMF, which is then converted (by glutathione *S*‐transferase activity) to a low molecular fluorescent thiol‐MF adduct. The rate of this adduct formation is expected to be proportional to the intracellular low molecular thiol level. Control experiments without LtP yielded no significant rates, whereas untreated control LtP showed strong fluorescence evolution. Incubation with EO components showed a decrease for all products, especially for Caryo, which might be a link to its mitochondrial effects (Figure 8).

## 4 DISCUSSION

EO prepared from Cuban C. ambrosioides L. plants was analyzed by gas chromatography/mass spectrometry showing that Asc, Car, and Caryo are the main components of the EO (Monzote et al., 2006). Independent studies on EO from Chinese C. ambrosioides L. also listed Asc, Car, and Caryo as detected components, though in different amounts (Chu et al., 2011). It was shown that the EO composition from C. ambrosioides L. can vary widely (Jesus et al., 2017; Soares et al., 2017). Our mechanistic studies of Asc, Car, and Caryo address only a part of pharmacological activities of our EO and of other EOs from C. ambrosioides L. Previous studies demonstrated that EO from C. ambrosioides L. was effective against *L. amazonensis* infections that cause cutaneous leishmaniasis in mice (Monzote, Pastor, Scull, & Gille, 2014). In a combinatorial study, it was shown that especially combinations of Asc:Car = 1:4 were most effective against lesion development in this cutaneous leishmaniasis model. In vitro studies using the *L. tarentolae* model showed that Asc is activated in *Leishmania* to a carbon‐centered radical species preferably by low molecular iron (Fe^2+^) from the labile iron pool in *Leishmania* (Geroldinger et al., 2017). This was corroborated by the finding that IC_50_ values for EO and Asc are strongly increased for *L. amazonensis* in the presence of iron chelators (Monzote, Garcia, et al., 2014). The selectivity of Asc/EO for *Leishmania* is based on their much higher labile iron pool compared with the labile iron pool of macrophage/monocyte host cells (Geroldinger et al., 2017). These data indicate that Asc is a nonmitochondrial radical generator in *Leishmania*. Bioenergetic studies suggested the mitochondrial ETC to be one of several direct or indirect molecular targets of main compounds of EO from C. ambrosioides L. in *Leishmania* parasites (Monzote, Garcia, et al., 2014). In mammalian mitochondria, except for Caryo, no direct effect of EO on mitochondrial ETC with respect to oxygen consumption was observed (Monzote et al., 2009).

In this study, the influence of EO main compounds on complexes of the mitochondrial ETC as possible targets for antileishmanial drugs was explored. Biological model systems have greatly facilitated the understanding of drug actions. In our study, besides whole LtP cells, mitochondrial fractions of LtP, ScY, and BH were used to study mitochondrial functions in LtP in comparison with other eukaryotic organisms, including mammals. *L. tarentolae* is a parasite of geckos of the species Tarentola annularis, belonging to the genus *Sauroleishmania* (Lainson & Shaw, 1987), and is not pathogenic for humans (Raymond et al., 2012). *L. tarentolae* has been widely used in pharmacological studies for (a) the screening of natural and synthetic products (Taylor et al., 2010), (b) the purification and characterization of proteins that are used for the screening of drugs with potential antileishmanial activity (Fritsche et al., 2007; Yakovich, Ragone, Alfonzo, Sackett, & Werbovetz, 2006), and (c) the amplification of genes involved in the resistance to certain antileishmanial drugs such as amphotericin B (Singh, Papadopoulou, & Ouellette, 2001) and sodium stibogluconate (Haimeur & Ouellette, 1998).

The in vivo efficiency of EO, Asc, and combinations of Asc/Car against cutaneous leishmaniasis is only partially reflected by in vitro viability assays. Although in LaP, IC_50_ values clearly demonstrate a high benefit of Asc; in LtP, IC_50_ values for Asc, Car, and Caryo are in a similar range. In a previous study, the major components of EO gave following IC_50_ values: Asc, 0.6 ± 0.06 μM; Car, 101 ± 30 μM; and Caryo, 22.2 ± 10.4 μM against LaP (Monzote, Garcia, et al., 2014), which are particularly different for Asc and Car. Although qualitatively the iron‐dependent activation of Asc/EO was confirmed in LaP (Monzote, Garcia, et al., 2014) and LtP (Geroldinger et al., 2017), the quantitative outcome of viability assays may strongly depend on the cell number to drug ratio, detection method and even on assay medium and premature activation of Asc in media. Our current studies explore this systematically. From the listed IC_50_ values for LtP in this work, it can be concluded that it is at least likely that all three major components (and also possibly nonstudied trace compounds) are involved in the antileishmanial action of EO.

Direct effects of drugs on mitochondria include inhibition of individual ETC complexes, transporters, and disturbance of the highly sensitive coupling of proton translocation with ATP production. Indirect effects, which do not occur immediately after drug exposure but after several hours, may be caused by drug metabolites (rather rare), the intrinsic pathway of apoptosis triggered by nonmitochondrial targets, lipid peroxidation of mitochondrial membranes, and other processes.

A hallmark of drug actions on mitochondria is inhibition of the mitochondrial ETC (Chan, Truong, Shangari, & O'Brien, 2005). Therefore, oxygen uptake by LtP and its inhibition by EO major compounds were studied (Figure 2). Both EO and Caryo produced a significant inhibition of LtP oxygen consumption. This suggests that Caryo in EO directly acts on mitochondria of *Leishmania*. In contrast, Car and Asc did not show immediate effects on LtP oxygen consumption. Although the assay time for measuring oxygen consumption was about 0.5 hr after drug exposure, viability measurements were performed after 48/72 hr of incubation indicating that our oxygen consumption assays address immediate inhibition effects.

To determine if Asc, Car, and Caryo also induced inhibition of individual ETC complexes in *Leishmania*, we compared compound actions in a crude LtP‐Mit with BH‐SMP (Table 1). The results observed herein demonstrated that no relevant activity of EO compounds on complexes I and II was found in *Leishmania*, whereas complex III was preferably inhibited by Caryo in both, LtP‐Mit and BH‐SMP, with slight preference for LtP‐Mit.

This observation raised the question whether the inhibition by Caryo is a universal effect on complex III of other species. Therefore, purified cytochrome *bc*_1_ complex from yeast was used, compared with *bc*_1_ complex from bovine heart, and the inhibition by compounds Asc, Car, and Caryo was assayed. Again, the strongest inhibition was caused by Caryo, whereas Asc and Car did not show a strong inhibitory effect. This confirmed the results from the mitochondrial fractions, that is, that Caryo directly targets complex III in different eukaryotic mitochondria. Because IC_50_ values of Caryo in both *bc*_1_ complexes were similar, it is likely that host cells and *Leishmania* are susceptible to Caryo. This is a possible mechanism how EO could influence the viability of host cell macrophages.

Due to the inherent relationship between generation of reactive oxygen species and respiratory chain inhibition, complex III was described as the main source of superoxide radicals in both mammals and *Leishmania* species (Carvalho et al., 2010; Dawson, Gores, Nieminen, Herman, & Lemasters, 1993; Garcia‐Ruiz, Colell, Morales, Kaplowitz, & Fernandez‐Checa, 1995). Mitochondrial inhibition is sometimes (depending on the site of inhibition) accompanied by increased mitochondrial superoxide production. In addition, impairment of the mitochondrial ETC by lipid peroxidation and protein oxidation may trigger mitochondria to produce more superoxide radicals. Also NADPH oxidases, P450 oxidases, or xanthine oxidases as well as low molecular weight iron and ascorbate (which are both present in *Leishmania*) are known nonmitochondrial superoxide radical sources. We studied superoxide production in LtP by the CMH/ESR method, which is highly specific for superoxide except the interference with Fe^3+^ (Dikalov, Skatchkov, & Bassenge, 1997). Therefore, these assays are usually performed in the presence of iron chelators to prevent this side reaction. This, however, has the limitation that under these assay conditions, we can only assess the effects of nonactivated Asc because Asc needs iron for its pharmacological action. In these experiments (Figure 3), both negative buffer control (lacking LtP) and positive control (in the presence of the complex III inhibitor AA) showed that the detection system is working. All three EO compounds slightly increased the superoxide radical formation in LtP in the assays time frame of about 15 min.

In the genus *Leishmania*, different low molecular weight thiol antioxidants are present: glutathione, trypanothione, cysteine, and ovothiol (Romao et al., 2006). In addition, *Leishmania* can synthesize ascorbate as a powerful antioxidant (Manhas, Anand, Tripathi, & Madhubala, 2014). Therefore, we assessed the redox state of thiols in the presence of Asc, Car, and Caryo by a CMFDA assay (Sarkar et al., 2009) resulting in a glutathione *S*‐transferase (Fyfe, Westrop, Silva, Coombs, & Hunter, 2012) catalyzed conjugation of MF to low molecular weight thiols. As shown, these findings are in line with superoxide radical measurements. Low molecular thiols are decreased by EO compounds upon 5 hr incubation (Figure 8) but possibly by different mechanisms.

In our study, Car shows neither mitochondrial inhibition nor mitochondrial uncoupling. Car is a phenol like the well‐known uncoupler 2,4‐dinitrophenol. However, the pKa value of 2,4‐dinitrophenol is around 4, whereas for normal (non‐nitro‐substituted) phenols like Car, the pKa value is around 10 (Rappoport & Frankel, 1967). This makes an action of Car as protonophore not very likely under physiological conditions. Others have shown that Car, upon prolonged incubation with superoxide radicals, forms rather stable ESR signals, which cannot be assigned to simple phenoxyl radicals (Deighton, Glidewell, Deans, & Goodman, 1993). The complex ESR signals suggest the presence of large conjugated systems, which could arise from oligomerized Car oxidation products. These trace products could have potential redox‐cycling properties causing additional harm to *Leishmania*. In our experiments, we used nonoxidized Car and therefore did not study these effects.

To address the situation including Asc activation, we performed experiments for Asc with prolonged incubation times and in the presence and the absence of iron chelators (Figure 4). The results clearly show that activation of Asc takes time and is strongly enhanced in the absence of iron chelators. In addition, it was demonstrated that Asc has no immediate direct effect on mitochondria but increases superoxide radical formation after activation.

This prompted us to study Asc effects on mitochondrial coupling (Figures 5, 6, 7). A major mitochondrial function in LtP is the generation of ATP, which can be impaired by inhibition of the ETC or by uncoupling of ETC from ATP synthase function. The latter is often triggered by breaking down the proton gradient across the inner mitochondrial membrane (driving ATP synthesis) by proton‐shuttling drugs or increased proton permeability of the inner mitochondrial membrane. Increased proton permeability can be mediated by radical‐triggered membrane lipid peroxidation. Coupling reflects the ability of mitochondria to adapt ATP production to ATP demands. Stress conditions such as treatment with antileishmanial drugs may directly or indirectly increase the ATP demand, usually triggering mitochondria to produce more ATP. Conversely, a decreased mitochondrial coupling impairs this stress response mechanism.

As shown, Asc has no immediate direct uncoupling effect, but after prolonged incubation (probably via Asc activation), mitochondrial coupling is impaired in LtP.

**Figure 9:**
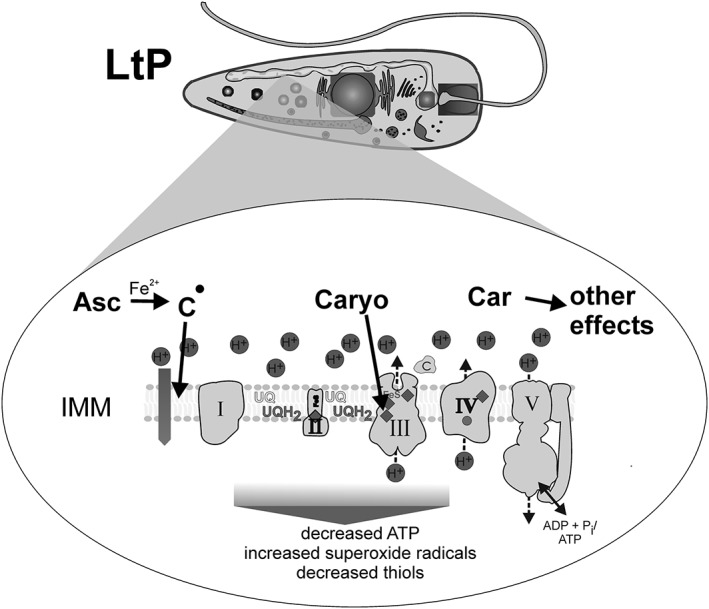
Scheme of the mitochondrial effects of major components of essential oil from Chenopodium ambrosioides L. on *Leishmania tarentolae* promastigotes (LtP). Asc = Ascaridole; Car = carvacrol; Caryo = caryophyllene oxide; IMM, inner mitochondrial membrane

EO from C. ambrosioides L. is a complex mixture with a variety of possible pharmacological mechanisms. In addition, there are numerous possibilities for pharmacological interactions as demonstrated for Asc and Car in a previous publication (Monzote, Pastor, et al., 2014). The three main compounds are responsible for some but certainly not for all effects (Figure 9). Among these compounds, only Caryo has direct inhibitory effects on complex III in *Leishmania* and other eukaryotic cells. Car has no inhibiting effects on ETC but similarly impairs LtP viability. Asc has the most complex mechanism. It has no direct inhibiting effect on mitochondrial ETC and no immediate uncoupling effect in LtP. However, upon activation by iron, Asc impairs mitochondrial coupling and triggers superoxide radical formation in LtP. This suggests that impairment of mitochondrial coupling in *Leishmania* by prolonged incubation with Asc (Figure 7) is not the primary mode of action but a downstream event of rather selective activation of Asc in *Leishmania* (Geroldinger et al., 2017). Further studies are required to elucidate possible synergistic effects of Car and Asc.

These findings suggest that Asc, Car, and Caryo mediate their leishmanicidal activity via different targets in mitochondria and in other parts of the cell and that different mitochondrial effects are seen after different times of exposure.

## CONFLICT OF INTEREST

All authors have no conflict of interest to declare.
